# An Unexpected Case of Cannabis-Induced Pancreatitis

**DOI:** 10.7759/cureus.13253

**Published:** 2021-02-10

**Authors:** Fiona Lubega, Anita Lwanga

**Affiliations:** 1 Internal Medicine, Saint James School of Medicine, The Quarter, AIA; 2 Internal Medicine, Samaritan Medical Center, Watertown, USA

**Keywords:** acute pancreatitis, cannabis, cannabinoids, tetrahydrocannabinol, thc

## Abstract

As cannabis becomes legalized in many jurisdictions around the world, its popularity is increasing. Despite the presence of several publications reporting cannabis-induced acute pancreatitis, the mechanism and pathophysiology of this entity are poorly understood. This case report highlights the course of a 28-year-old male who presented to the hospital with complaints of abdominal and back pain. Because his lipase was elevated, he met the revised Atlanta criteria for a diagnosis of mild acute pancreatitis. After excluding more common causes, it was determined that his pancreatitis was likely due to cannabis use. This case adds to the body of research reporting on cannabis-induced acute pancreatitis. In the future, we hope to see strict diagnostic criteria and further research to elucidate the mechanism and pathophysiology of this entity.

## Introduction

Cannabis is the most widely used recreational drug worldwide, with over 4% of the world’s population using it annually [1-2]. It has been used to treat nausea and vomiting secondary to chemotherapy, abdominal pain, cancer-related pain, irritable bowel syndrome, and to reduce the symptoms of acute inflammatory bowel disease [3]. Paranoia, psychosis, infertility, and visual changes are the documented side effects of cannabis use [1-2,4].

Common causes of acute pancreatitis include gallstones, alcohol abuse, smoking, hypertriglyceridemia, infections, trauma, drugs, malignancy, scorpion stings, hypercalcemia, and endoscopic retrograde cholangiopancreatography (ERCP) [1-2,5-6]. Opioids, angiotensin-converting enzyme (ACE) inhibitors, macrolides, nonsteroidal anti-inflammatory drugs (NSAIDs), diuretics, statins, and cannabis have been associated with acute pancreatitis [2,5]. While prescription medications account for 2% of cases of acute pancreatitis, cannabis use accounts for even fewer cases [2].

In the first reported case of cannabis-induced pancreatitis, which was published by Grant and Gandhi, a challenge posed to clinicians was the patient’s hesitation to disclose his cannabis use because of issues surrounding the legality of the substance [1]. Restrictions on cannabis use have since been lifted; this raises the possibility for further exploration of this topic [1]. We report a rare case of cannabis-induced pancreatitis, which we hope will add to the body of research supporting the presence of this entity.

## Case presentation

A 28-year-old male presented to the hospital with complaints of nausea, vomiting, and severe lower abdominal pain that radiated to his back. He denied having fevers, chills, constipation, or diarrhea. The patient had a medical history of diverticulitis and migraines. He did not have a family history of medical problems, did not have allergies, and did not take any medications. He did not use alcohol or tobacco products but admitted to using cannabis. 

The patient’s temperature was 99.2°F, heart rate was 77 beats per minute, respiratory rate was 20 respirations per minute, and his blood pressure was 142/83 mmHg. His abdomen was flat, he did not have Cullen's sign or Grey Turner’s sign, bowel sounds were hypoactive, and he complained of tenderness with palpation of the lower abdomen. The rest of his physical exam was unremarkable. His white cell count was 14,700/µL and a serum lipase was 1,022 U/L. The rest of his complete blood count, serum electrolytes, serum calcium, serum creatinine, serum urea, aspartate aminotransferase, alanine aminotransferase, alkaline phosphatase, lactate dehydrogenase, serum bilirubin, cholesterol, and triglycerides were within their reference range. His serum ethanol was negative, but his urine drug screen was positive for delta-9-tetrahydrocannabinol (THC) and opiates. Both the computed tomography (CT) scan of the abdomen and pelvis and ultrasound of the liver and gallbladder were unremarkable. 

The patient’s complaints of abdominal pain and presence of lipase that was elevated more than three times the upper limit of normal, met the revised Atlanta Criteria to diagnose mild acute pancreatitis [7]. The patient’s medical and social history ruled out tobacco abuse and prescription medications as possible causes but raised the possibility of cannabis-induced pancreatitis. The blood tests and imaging studies ruled out gallstones, alcohol, IgG4-related disease, or hyperlipidemia as possible causes of acute pancreatitis. His urine drug screen was positive for opiates because he received morphine in the emergency room to manage his pain prior to collecting a urine sample. The presence of THC in the urine drug screen further affirmed the possibility that the pancreatitis was due to cannabis use.

To help confirm the possibility of cannabis-induced pancreatitis we used the Naranjo Nomogram for Adverse Drug Reaction Assessment. The patient’s Naranjo score was 6, which corresponds with a rating of probable adverse drug reaction. This confirmed cannabis as the probable cause of his acute pancreatitis. Initially, the patient was instructed not to eat and received intravenous fluids along with intravenous Toradol and morphine to manage his abdominal pain. His abdominal pain improved prior to discharge from the hospital and he was instructed to follow up with his primary care provider. Fortunately, he has not been readmitted to the hospital.

## Discussion

Cannabinoid receptor 1 (CB1) and cannabinoid receptor 2 (CB2) are the two main types of cannabinoid receptors in the human body [3,6,8-9]. While CB1 is found primarily in the nervous system, CB2 is found primarily in the gastrointestinal tract and immune cells [4,8]. Out of approximately 113 cannabinoids that have been identified, delta-9-tetrahydrocannabinol (THC), and cannabidiol (CBD) are best understood [10]. THC is a partial CB1 and CB2 agonist; it has a higher affinity for the CB1 receptors in the brain, which is responsible for the euphoric feeling expressed by users and may also cause anxiety and psychotic disorders [8,11]. On the other hand, CBD, which has antipsychotic, antianxiety, anti-nausea, antibacterial, antifungal, and anti-inflammatory effects can mitigate the effects of THC [8]. In the pancreas, both CB1 and CB2 receptors are weakly expressed in the islets of Langerhans [2-3,8]. When the pancreas is inflamed, the expression and activity of both CB1 and CB2 receptors are increased [8]. Wargo et al. proposed that agonism of the CB1 may play a role in the mechanism of acute pancreatitis, but to the best of our knowledge, this has not been confirmed in human studies [2]. Beyond this, little is known about how cannabinoids interact with receptors in the pancreas to cause acute pancreatitis [1,3-6]. It is possible that cannabis-induced pancreatitis may be dose and time-dependent, as seen in chronic and heavy smokers where symptoms of acute pancreatitis resolve with cessation of cannabis [1-3].

Cannabis-induced pancreatitis is considered a diagnosis of exclusion [4]. Beyond the information the patient provides, it is difficult to confirm a diagnosis of cannabis-induced pancreatitis because there are no strict guidelines to confirm the diagnosis [12]. Several case series and case reports have utilized the Naranjo Nomogram for Adverse Drug Reaction Assessment to support the diagnosis of cannabis-induced pancreatitis in their patients [2,8-13]. It may be useful for future guidelines on managing and treating cannabis-induced pancreatitis to incorporate the Naranjo score in the diagnostic criteria. With the exception of a case report where a patient presented with cannabis-induced pancreatitis at 48 years of age, other cited reports featured a pattern of symptom presentation in patients in their 20s, similar to our patient [1-2,8,12]. The 48-year-old patient presented with multiple comorbidities, including diabetes, hyperlipidemia, hypertension, and gastroesophageal reflux, which was in contrast to most of the younger patients, including our patient, who had unremarkable medical histories [12]. The most common finding in the featured cases was a dose-dependent correlation between cannabis use and symptom presentation [1,8]. One of the limitations of this case report is that we did not obtain information about our patient’s frequency amount of cannabis use. In one report, a CT finding in a 25-year-old patient showed an edematous pancreas with peripancreatic fat stranding and fluid collection, which was in contrast to our patient’s unremarkable CT finding [8].

## Conclusions

Cannabis is an unusual cause of acute pancreatitis. There is a paucity of information about how cannabinoids interact with receptors in the pancreas to cause acute pancreatitis. It is our hope that further research will be undertaken in this area, to facilitate an in-depth understanding of this condition.
